# Salivary and blood plasma oxytocin after oxytocin injection and during machine milking in dairy cows

**DOI:** 10.3168/jdsc.2025-0761

**Published:** 2025-06-03

**Authors:** Olga Wellnitz, Benjamin Jenni, Natascha Stoffel, Samantha Weber, Selma Aybek, Rupert M. Bruckmaier

**Affiliations:** 1 Veterinary Physiology, Vetsuisse Faculty, University of Bern, 3012 Bern, Switzerland; 2 Faculty of Science and Medicine, University of Fribourg, 1700 Fribourg, Switzerland; 3 Graduate School of Health Sciences (GHS), University of Bern, 3012 Bern, Switzerland; 4 University of Zurich, Psychiatric University Hospital Zurich, Department of Psychiatry, Psychotherapy and Psychosomatics, 8032 Zurich, Switzerland

## Abstract

Oxytocin (OT), the peptide hormone whose release from the pituitary is essential to induce milk ejection, has been measured in blood plasma of dairy cows for decades. In the present study we tested the possible use of saliva instead of blood sampling to record OT release by using a commercially available ELISA for salivary OT measurement both in saliva and blood plasma. Measurements were performed to detect the expected OT increase in response to intravenous OT injections and during machine milking. Three dairy cows were injected with increasing dosages (0.5, 1, 5, and 10 IU i.v.) of OT, and blood and saliva samples were repeatedly taken during 1 h after injection. Furthermore, 5 cows were milked and blood and saliva samples were taken during and until 20 min after milking. To verify the expected release of OT during milking, we used our established RIA for measurements in plasma in parallel to ELISA measurements. Our results showed that the commercial ELISA kit used was able to detect the OT concentrations in both saliva and plasma. The massive increase of OT concentration after OT injection and during milking could be detected in plasma with both RIA and ELISA. However, we did not observe any change of salivary OT within the duration of the experiments. Our results showed that short-term increases in blood plasma OT, such as in response to OT injection or milking, were not reflected by increasing salivary OT concentrations.

## Highlights

• Oxytocin can be measured in dairy cow saliva at low levels with RIA or ELISA.
• After injection and during milking, OT concentrations increase in blood plasma but not saliva.
• The commercial ELISA used for salivary OT can be used to measure blood plasma OT.

## Graphical Abstract

**Figure:**
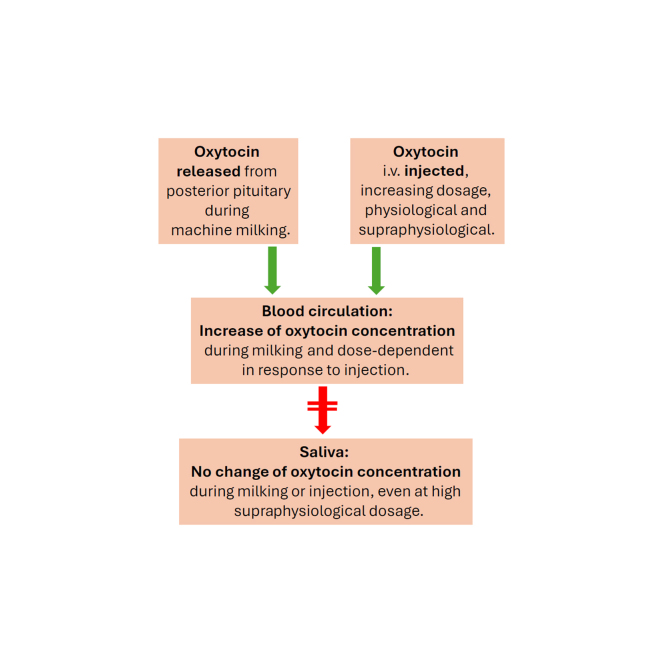
**Summary:** In this study, we tested the possible replacement of blood with saliva sampling to measure oxytocin (OT) by using a commercial enzyme-linked immunosorbent assay (ELISA) for salivary OT measurement both in saliva and blood plasma. Cows were either intravenously (i.v.) injected with different doses of OT or machine milked. Blood and saliva samples were taken. To verify the expected release of OT during milking, we used our established radioimmunoassay (RIA) for measurements in plasma in parallel to ELISA measurements. Our results showed that the commercial ELISA kit used was able to detect OT concentrations in both saliva and plasma. The expected increase of OT concentration after injection and during milking could be detected in plasma with both RIA and ELISA. However, salivary OT remained unchanged. Our results showed that shortterm increases in blood plasma OT, such as in response to OT injection or milking, are not reflected in increasing salivary OT concentrations.

Oxytocin (**OT**) is a peptide hormone synthetized by hypothalamic neurons and released into the peripheral blood circulation by the posterior pituitary to induce contraction of specialized smooth muscles of the uterus myometrium during parturition and of mammary myoepithelial cells during milk ejection. In addition, OT has been demonstrated to be released within regions of the central nervous system to control various emotional processes such as maternal behavior or pair bonding (Neumann, 2008). Because the plasma concentration of OT is in the lower picogram per milliliter range, reliable measurement of its concentration is relatively complicated. Only in the late 1970s were adequate immunological test systems (RIA) with sufficiently high sensitivity developed (Gorewit, 1979; Schams et al., 1979). In various species, including lactating farm animals, OT measurement has served as a physiological marker to characterize and understand the processes related to milk ejection in dairy cows during milking and suckling (Bruckmaier and Blum, 1998; Wellnitz and Bruckmaier, 2001). More recently, commercial ELISA test kits have been developed for measuring several hormones, including OT, in animals and humans, to avoid the use of radioactive components in the laboratory. In addition, ELISA for OT determination are available not only for measurements in blood plasma but also in saliva from several species, including humans (Rana et al., 2023), swine (López-Arjona et al., 2020; Hall et al., 2021), and cattle (López-Arjona et al., 2021). However, for dairy cows it has not been shown if the OT concentration in saliva mirrors the concentration in blood during short-term changes such as milk ejection and, if so, in what ratio. In comparison to blood samples, the collection of saliva samples in dairy cows is easy, noninvasive, and well established for hormones such as cortisol (Schwinn et al., 2016). However, changes in these hormones in saliva occur with some delay compared with plasma (Schwinn et al., 2016). The aim of this study was therefore to test the hypothesis that plasma OT concentrations are reflected in saliva. It should be clarified whether the salivary OT increases to the same extent as in the blood and, if so, with what time delay, to determine the best possible time point to collect saliva samples for characterization of the OT release related to milk ejection in dairy cows.

The experiment was approved by the Veterinary Office of the Canton of Fribourg (Granges-Paccot, Switzerland; authorization no 2023-23-FR). To test the parallelism of OT in saliva to low and high OT concentrations in blood, 3 Holstein dairy cows in mid lactation were intravenously injected with OT at increasing doses through a catheter in a jugular vein (0.5, 1, 5, and 10 IU OT; Graeub, Switzerland) diluted in 5 mL of saline solution, with 2 h between the different doses. Blood samples were taken from a catheter in the contralateral jugular vein immediately before each OT injection, then every 2 min for 10 min and every 10 min up to 60 min after each OT injection. Starting 1 min before blood collection, saliva samples were collected continuously for 10 min and then every 10 min for 2 min of collection. Each collection sponge stayed in the mouth of the cow for 1 min before it was exchanged with a fresh sponge.

In addition, saliva and blood sampling was performed during machine milking of 5 Holstein dairy cows in mid lactation. Milking was performed at the routine milking time using the milking equipment normally used to milk these cows. Immediately before and then every 2 min after the start of a 1-min udder prestimulation and attachment of the milking cluster, and then every 10 min until 20 min after the start of milking blood samples were taken through a jugular vein catheter inserted at least 1 h before milking. Saliva samples were collected continuously for 20 min starting one minute before the first blood sampling, while the collections sponge (described later in this section) was changed every minute. Irrespective of the sampling, the milking cluster was removed at cessation of milk flow after udder emptying.

All blood samples (10 mL) were treated with Na_2_-EDTA to avoid coagulation. Samples were stored on wet ice until centrifugation. Plasma was obtained by centrifugation (3,000 × *g*, 20 min, 4°C) and stored at −20°C until analysis.

Saliva samples were collected by keeping the sponge of Salivette collection tubes (Sarstedt) held by a clamp in the mouth of the cow for 1 min. Tubes were kept on wet ice until processing (within 2 h), by centrifugation at 4°C and 3,000 × *g* for 20 min and storage at −20°C until analysis. Samples from 2 sponges each (2 min) were pooled for OT extraction to achieve sufficient volume of saliva (minimum 1 mL).

The OT in saliva and plasma was measured using an ELISA kit including extraction, which was designed for measurements in saliva (Enzo Life Sciences Inc., #ADI-901-153A) according to the manufacturer's instructions. Saliva samples were concentrated 4-fold by using 1 mL of sample for extraction to be reconstituted in 250 μL of buffer. To validate the ELISA's ability to measure OT, we spiked both plasma and saliva samples with different amounts of OT before extraction. We observed a recovery rate for OT of 85%–90%. Intra- and interassay CV of the ELISA were 5% and 11%, respectively.

In addition, plasma OT during machine milking was measured by our established RIA according to Schams (1983) using 1 mL of plasma for extraction. The RIA measurement with an established extraction step was used for OT measurement in plasma samples during milking to verify the expected OT increase as measured during milking in previous studies (Schams, 1983; Bruckmaier and Blum, 1998). Most of the saliva samples did not provide enough saliva for OT extraction for RIA measurement. Therefore, OT was only measured by RIA in some saliva samples (data not shown) and showed comparable concentrations as measured by ELISA.

Due to the basic character of the study and low number of individuals, the results are only presented descriptively. Results of measurements in saliva and plasma after OT injection and during milking of individual cows are shown in Figure 1, Figure 2. Oxytocin was measurable in both saliva and plasma using the commercial ELISA kit. This was expected and in line with a previously published study (Lürzel et al., 2020). Comparison of OT concentrations in saliva with those in plasma showed higher baseline concentrations in saliva than in plasma. In the present study, plasma OT concentrations increased immediately after OT administration, with peak concentrations in the first sample after injection (after 2 min). Highest concentrations of up to >250 pg/mL were achieved after the administration of 10 IU of OT (Figure 1B). However, an increase of OT in saliva was not detectable within the 1 h of sampling in all cows with all doses of exogenous OT administration, relative to baseline salivary concentrations. This was independent of the basal OT concentrations in saliva.

**Figure 1:**
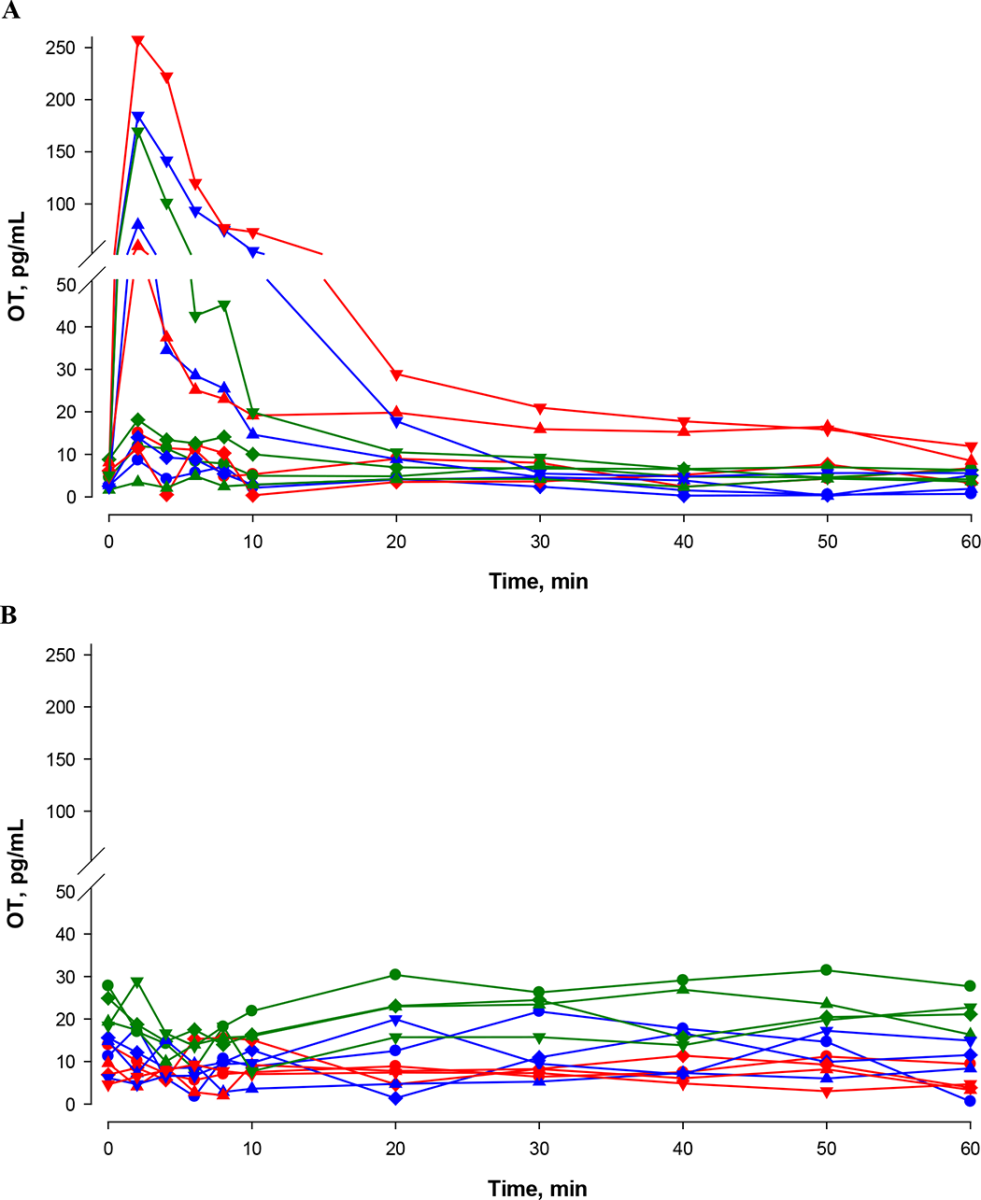
Oxytocin (OT) concentrations in blood (A) and in saliva (B), measured by ELISA, of 3 cows (red = cow 1; blue = cow 2; green = cow 3) before and after intravenous injection of 0.5 (•), 1 (♦), 5 (▴), and 10 (▼) IU of OT.

**Figure 2:**
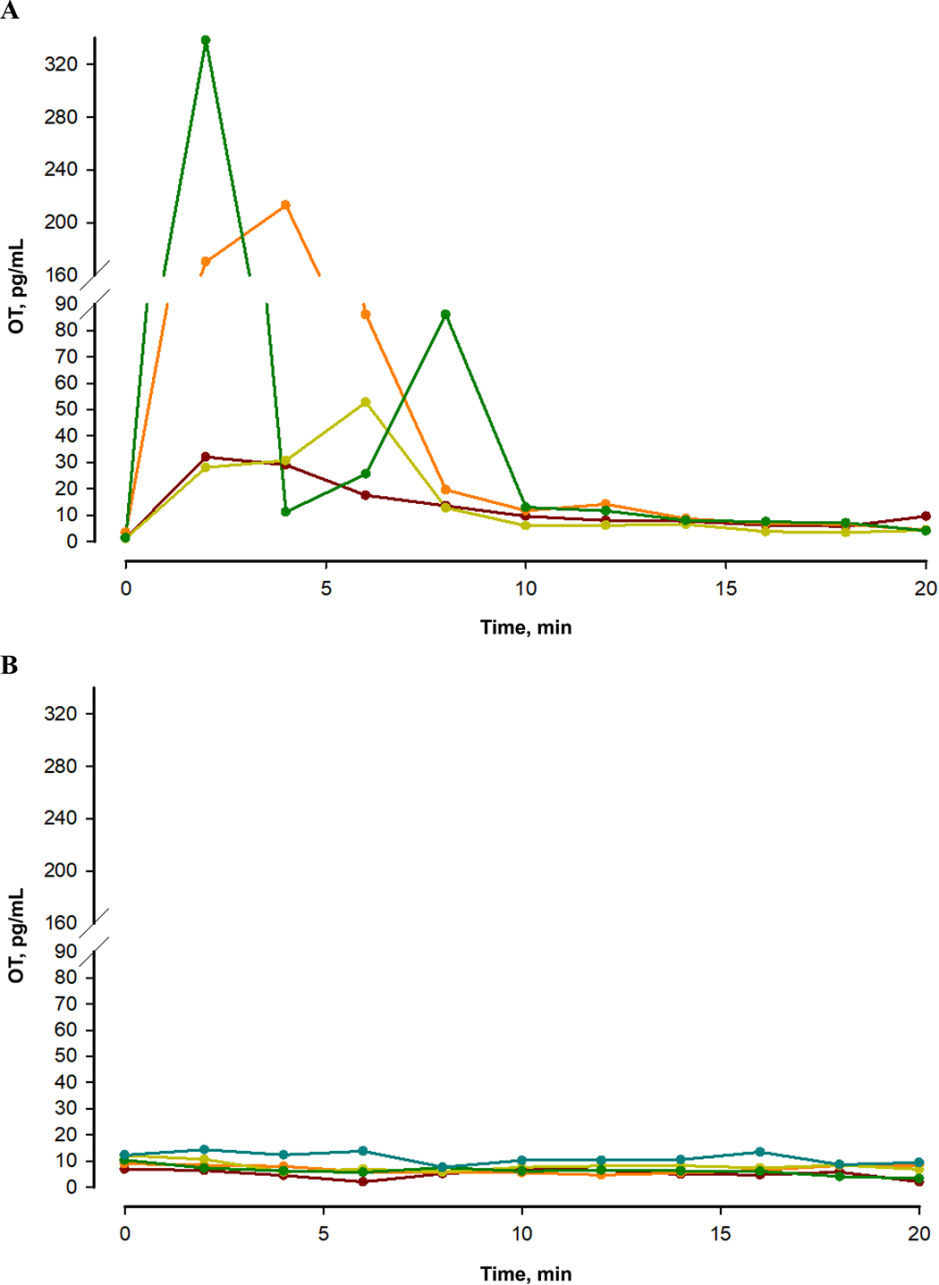
Oxytocin (OT) concentrations in blood plasma (A; measured by RIA) and in saliva (B; measured by ELISA) of 5 cows (brown = cow 1; orange = cow 2; olive green = cow 3; dark green = cow 4) after the start of milking. For technical reasons, the blood plasma samples of cow 5 are missing.

Our study showed that short-term increases in OT, such as in response to OT injection or milking could be measured in plasma but not in saliva. To our knowledge it has not been demonstrated how OT is transported from blood into saliva. In the brain of mice, an active transport from the blood has been demonstrated (Yamamoto et al., 2019). Furthermore, OT can reach the central nervous system after inhalation (Quintana et al., 2018). In the salivary gland, different hormones like steroids, but also proteo-hormones like insulin, are transferred from blood into the saliva through different mechanisms, including active transport, passive diffusion, or expression and secretion (Gröschl, 2008). Because OT is not soluble in lipids, it is assumed that it is transported from the blood through tight junctions between the salivary epithelial cells as seen with other hormones (Vining et al., 1983). Therefore, we expected an increase of OT in saliva after a pronounced increase of OT in blood. However, based on our results a diffusion through tight junctions is not possible. Because OT has a half-life of ∼2 to 3 min in cows (Belo and Bruckmaier, 2010), we did not assume that an increase in saliva would occur later than 1 h after injection, and therefore, no more samples were taken after this time.

Previous studies have shown that on the day of parturition, when high concentrations of OT are found in plasma, increased levels of OT can be measured in saliva of cows (López-Arjona et al., 2021). Therefore, in the present study, samples were also collected during milking (Figure 2), when high OT plasma concentrations are observed for short periods of time, to exclude a different transfer and measurability between exogenously administered and endogenously released OT in saliva. During milking, the expected considerable increase of OT in plasma was confirmed by RIA measurement (Figure 2 A). It was comparable to previously published results (Bruckmaier and Blum, 1998). However, an increase of OT was not detectable in the salivary samples that were taken in parallel (Figure 2 B). Although the number of cows included in this experiment was low, the results clearly demonstrate that the concentrations of OT in saliva do not correlate with the concentrations of OT in plasma.

A poor correlation between plasma and saliva concentrations of OT has been described in humans (Martin et al., 2018). Furthermore, it has been discussed that salivary OT concentrations do not accurately reflect plasma OT concentrations and that salivary OT and enzyme immunoassay-based plasma OT correlate only moderately, ranging from r = 0.41 to r = 0.59 (Martin et al., 2018). With our study, we demonstrate that this phenomenon is similar in cows and that OT cannot simply traffic from blood into saliva. From the low correlation of salivary and plasma OT, it has been suggested that OT might not be a suitable biomarker when measured in saliva (Horvat-Gordon et al., 2005). Remarkably though, OT concentrations in the brain of humans correlated more with salivary OT than with OT measured in plasma (Carter et al., 2007). Because we detected higher basal concentrations of OT in saliva in the cow, and, to our knowledge, there is no evidence that OT can be produced in salivary glands, it is possible that the OT in saliva reflects OT concentrations in the brain and is thus not subject to fluctuations in the blood due to the release from the pituitary gland. It is possible that the differences in peripheral and central OT concentration reflects both the physiological processes, such as the blood-brain-barrier, but also functionally distinct effects that need to be considered. Hereby OT in the brain might serve the purpose of emotional-social changes in behavior (Weber et al., 2024), whereas OT in plasma relates to the parturition and lactation events (Neumann and Landgraf, 2012). In fact, in lactating monkeys, OT levels in the cerebrospinal fluid were independent of the lactation and suckling stimulus. Additionally, it was observed that plasma OT increases in response to the nursing stimulus (Amico et al., 1990). It is possible that there are different and unknown pathways of OT transfer and function per biofluid, and this may explain our observations in the dairy cow.

In conclusion, our study demonstrated that large short-term increases of OT concentrations in plasma, by exogenous injection or endogenous release from the pituitary during milking, is not reflected in the saliva of dairy cows. Therefore, the measurement of OT in saliva in cows cannot be used for evaluation of sufficient OT release during milk removal and to characterize milk ejection during milking. However, our data suggest that commercially available ELISA kits may be able to replace RIA measurements of OT in blood and saliva of cows.
